# Health, Lifestyle, and Gender Influences on Aging Well: An Australian Longitudinal Analysis to Guide Health Promotion

**DOI:** 10.3389/fpubh.2014.00070

**Published:** 2014-07-02

**Authors:** Hal Kendig, Colette J. Browning, Shane A. Thomas, Yvonne Wells

**Affiliations:** ^1^Centre for Research on Ageing, Health and Wellbeing, Australian National University, Canberra, ACT, Australia; ^2^ARC Centre of Excellence in Population Ageing (CEPAR), Monash University, Melbourne, VIC, Australia; ^3^School of Primary Health Care, Monash University, Melbourne, VIC, Australia; ^4^Australian Institute for Primary Care and Ageing, La Trobe University, Melbourne, VIC, Australia

**Keywords:** healthy aging, life style factors, gender, self-rated health, functional independence, psychological well-being, prospective design

## Abstract

A primary societal goal for aging is enabling older people to continue to live well as long as possible. The evidence base around aging well (“healthy,” “active,” and “successful” aging) has been constructed mainly from academic and professional conceptualizations of mortality, morbidity, functioning, and psychological well-being with some attention to lay views. Our study aims to inform action on health promotion to achieve aging well as conceptualized by qualitative research identifying what older Australians themselves value most: continuing to live as long as possible in the community with independence in daily living, and good self-rated health and psychological well-being. Multivariate survival analyses from the Melbourne longitudinal studies on healthy aging program found that important threats to aging well for the total sample over a 12-year period were chronological age, multi-morbidity, low perceived social support, low nutritional score, and being under-weight. For men, threats to aging well were low strain, perceived inadequacy of social activity, and being a current smoker. For women, urinary incontinence, low physical activity and being under-weight were threats to aging well. The findings indicate that healthy lifestyles can assist aging well, and suggest the value of taking gender into account in health promotion strategies.

## Introduction

Several decades of research have examined bio-psycho-social influences on positive experiences of aging (aging well) in contrast with the disease or degenerative focus dominant in bio-medical paradigms of aging. There has been increasing public and policy recognition of the life goals of older people themselves and growing recognition that aging experiences are potentially improvable through constructive individual and social action. Important milestones in these developments included the World Health Organization (WHO) Active Aging Policy Framework (1) and healthy and active aging initiatives in Canada, the United Kingdom, the United States, and Australia (2–5). Further waves of research and policy action have emerged in Europe (6) where 2012 was designated the European year of Active Aging. In the US, the Centers for Disease Control and Prevention (CDC) have focused on physical activity as a marker of active aging (7).

Our Australian longitudinal investigation of the multi-dimensional outcome, aging well [the Melbourne longitudinal studies on healthy aging: MELSHA; (8)] was developed during the 1990s with support initially from the Victorian Health Promotion Foundation (VicHealth). The Foundation had developed a population group approach to health promotion, rather than a disease or risk factor approach, inclusive of older as well as younger people (9). Before funding our Health Status of Older People (HSOP) program (1993–1998; the first stage of MELSHA), the Foundation convened an advisory group of health professionals, policy and program managers, and community advocates, who worked with the multi-disciplinary research team to develop a research plan that aimed to inform health promotion strategies. The advisory group aimed to build an evidence base to promote the independence and well-being of older people, focusing on opportunities for prevention and improvement, identifying “target groups” for action, and considering implications for health services (10). The current paper reports findings concerning predictors of aging well over a 12-year period based on the MELSHA program. We focus on socio-economic, health, gender, and lifestyle influences that are potentially amenable to improvement throughout the life course in order to inform the development of interventions and programs for older people.

### Conceptualizations of aging well

While designing the HSOP program, the research team concurrently conducted exploratory, qualitative investigations to elucidate older people’s own life goals and explore the significance of health for maintaining their identity and continuity of self (11). We found that their health ideals (subsequently confirmed in our baseline HSOP survey) centered around keeping active, feeling well, having a positive outlook, and maintaining independence as well as an absence of disease or functional limitations. Informants focused on the importance of being able to “continue to go about your everyday life” as an important aspiration for aging well. Further, major gender differences were found; for example, men were relatively more concerned with health for continuing performance capacities while women viewed health more as a means to continue their social connectivity. This latter finding highlights the need to examine gender effects in the present paper. A later Australian study found that loss of independence and entry to residential care are two of the major fears for the future among older people in the community (12).

A recent international review of qualitative studies similarly found that laypersons had complex conceptualization of “successful aging” extending far beyond physical health to include psychosocial components – notably social engagement, attitudes, and well-being (13). A review of “healthy aging” concepts across different cultures concluded that lay views were more complex and multi-dimensional than were academic views; for older people healthy aging is more than physical, mental, and social functioning (14).

An extensive and diverse quantitative literature has emerged on successful, healthy, active, and productive aging, including their definitions, extent, and predictors (15–22). Central to this body of work was the landmark MacArthur project that launched “aspirational” studies intended to identify ways to achieve “successful” rather than “usual” aging (23). Successful aging was defined as a multi-dimensional concept with the three components of “avoiding disease,” “engagement in life,” and “maintaining high cognitive and physical function.” Empirical investigations of this approach have set high thresholds for “success” with the consequence that “usual” aging is inevitably viewed as “unsuccessful.” As noted by Depp et al. (24), more attention has been paid to the physical health dimensions of successful aging than to emotional and cognitive health. Self-rated health has been used as a proxy for successful aging in a number of studies (25) while psychological studies have focused on well-being and mental health (24, 26, 27). Our conceptualization and measurement criteria for “aging well,” as developed in this article, recognize that the majority of older people view themselves as aging well through most of later life, notwithstanding age-related health and social transitions.

### Predictors of aging well

Our initial review of the international literature when planning this research [see Ref. (28)] found a pre-dominance of cross-sectional studies, thus excluding those who had died or moved to residential care, and shedding little light on the potentially modifiable causal influences on aging well such as health behaviors and lifestyles. Our later review of the more powerful longitudinal evidence base, found a pre-dominance of epidemiological studies focused on single health outcomes such as physical, cognitive, or mental functioning, or on morbidity or mortality (29). Some of the studies reviewed confounded predictor and outcome variables. Few have taken into account the biases introduced by selection effects attributable to differential rates of survival, entry to residential care, and non-response.

A number of longitudinal investigations have examined risk and protective factors for various outcomes related to aging well in old age. In an early review, Stuck et al. (30) concluded from prospective studies that the most significant risk factors for functional decline were cognitive impairment, depression, disease burden, under- or over-weight, lower limb functional limitation, low social activity, low physical activity, poor self-perceived health, no alcohol use, smoking, and vision impairment. Later studies and reviews have confirmed the role of behavioral and social factors such as not smoking, physical activity, normal weight, moderate alcohol use, and social integration in physical functioning, cognitive functioning, and mortality in older people (17, 31, 32). A 19-year prospective study of mortality among people in the United States aged 25 years and older (33) found that low physical activity and smoking remained significant, after controlling for confounding factors, while neither body mass index (BMI) nor education were significant after controlling for health behaviors.

A relatively new line of research has been examining social determinants of health and successful aging. A 17-year follow-up of the Whitehall II study (34) found that successful aging – as indicated by cognitive capacities, absence of disease, and good functional health – was predicted by socio-economic factors in mid-life and a range of key health behaviors earlier in life, including diet and exercise, as well as work support for men. Cross-sectional analyses of the baseline data from our Health Status of Older People study of older Australians (35) found that former occupation, education, income, and home ownership were independently related to physical and social activity and, further, that a continuing capacity to remain independent enabled high levels of well-being notwithstanding the presence of illness and pain (36). Research from the English longitudinal survey on aging (ELSA) has identified substantial social class inequalities in the onset of illness and survival (37).

In sum, there is an accumulating body of longitudinal research examining the associations between older people’s characteristics and behaviors with their subsequent experiences in terms of various measures of aging well. Research has been limited, however, by difficulties distinguishing between causal influences and outcomes as the same measures are sometimes used as both independent and dependent variables (38). In addition examination of single outcomes and/or single risk or protective factors may be confounded by other factors not included in the analyses. Physical and mental/emotional health components of aging well are rarely examined together as a multi-dimensional aging well outcome. Finally, results are seldom specified in terms of gender and other social groups.

### Study purpose

In this article, we aim to identify key influences on “aging well” as a holistic, multi-dimensional concept of health, and well-being aligned with older people’s own aspirations for aging recognizing the influence of gender [see also Ref. (16)]. Our broad model follows Maslow (39) and later Rowe and Kahn (23) in conceptualizing a hierarchy of inter-related aspects of goals for aging: first, staying alive (survival); and second (if alive) staying in the community (as opposed to living in residential aged care), and third (if in the community) continuing to live independently with good physical and psychological health. In a previous paper, we examined predictors of entry to residential aged care (not living in the community) over a 12-year period from the MELSHA data (40) and found that the significant independent predictors were older age, dependence in instrumental activities of daily living (IADL), cognitive impairment, under-weight BMI, and low social activity. In the present paper, we focus on aging well as an outcome and it is expected that different predictors will emerge for those who continue to live and age well in the community as compared to those who enter residential aged care.

To be classified as aging well in this paper our participants need to continue to live in the community independently (not in residential aged care) with good physical and psychological health. In this paper we address the following research questions:

1. What is the relative importance of baseline threats to aging well and protective factors in enabling older people to continue to age well through a 12-year period?
2. Are aging well outcomes and associated factors different for men and women?

## Materials and Methods

The information source for the analysis is the series of data collections that form the MELSHA program funded initially by VicHealth and subsequently by the National Health and Medical Research Council. The background and methods of the study have been reported in greater detail elsewhere (8, 10). The 1994 HSOP baseline survey included interviews with 1000 people living in private dwellings in metropolitan Melbourne, aged 65 years and over in 1994. The survey did not include people who were living in non-private accommodation (including residential aged care); those who could not speak basic English (11.3% of the sample frame); nor those who could not be interviewed for health reasons. Excluding these “out of scope” categories, the response rate for the initial interview was 70%, yielding a sample representative of older people living in the community in Melbourne at the time, apart from those too ill to be interviewed and non-English speaking people.

Over the 12-year period of the study analyzed to date, respondents in the baseline survey were followed up biennially in telephone interviews and by mail in the intervening years. Where respondents could not be contacted directly, the tracing procedures relied primarily on next of kin or other individuals volunteered by respondents as key contacts at the time of the baseline interviews. Death records were checked for individuals who were known to have died as well as for those who otherwise could not be contacted. While the MELSHA data collection continued until 2010 the number of participants in later waves beyond 2006 was significantly diminished reducing statistical power. We therefore focused on the 1994–2006 period of the study where the number of surviving participants was sufficient for our analyses.

The baseline data collection conducted in respondents’ homes included a face-to face interview, a brief physical assessment, and a self-completion instrument, which was returned later by mail. The questionnaire drew on a range of validated health status surveys available at the time and new measures on older people’s own views developed out of our earlier qualitative research (10). Topic areas included health-related actions, functional health, priority health conditions, quality of life, and service use. Table 1 footnotes specify measures for items examined in this analysis.

**Table 1:** **Baseline (1994) characteristics of participants by gender (*n* = 1000)**.

Characteristic	Total sample	Men	Women	*p* Value
**SOCIO-DEMOGRAPHIC**				
Gender (%)		47	53	
Age (mean)	73.4	72.6	74.1	<0.001
**MARITAL STATUS (%)**				
Never married	5	4	5	<0.001
Divorced/separated	5	4	6	
Married/living together	58	80	39	
Widowed	32	12	50	
Education (mean)^a^	1.32	1.51	1.15	<0.001
Income score (mean)^b^	4.93	5.42	4.50	<0.001
Living alone (%)	34	15	50	<0.001
Living children (%)	12	10	14	0.082
**COUNTRY OF BIRTH (%)**				
Australian	74	71	77	
English	10	9	11	
European	12	15	9	
Other	4	4	3	
**HEALTH**				
Tally of medical conditions (mean)	3.84	3.64	4.02	0.016
Self-rated health (mean)^c^	1.55	1.49	1.60	0.128
IADL dependent (%)^d^	18	10	24	<0.001
Depressed (%)^e^	7	5	9	0.028
Falls last year (%)^f^	10	6	12	0.002
Pain (mean)^g^	2.55	2.37	2.71	0.001
Urinary incontinence (mean)^h^	0.24	0.19	0.29	<0.001
Cognitive impairment score (mean)^i^	0.87	0.81	0.91	0.110
**LIFESTYLE**				
Strain (mean)^j^	4.46	4.50	4.42	0.112
Restful sleep (mean)^k^	3.65	3.72	3.58	0.002
Total physical activity (mean)^l^	8.64	8.25	8.99	0.007
Nutrition score (mean)^m^	14.02	14.25	13.82	0.048
Perceived social activity adequacy (mean)^n^	1.82	1.84	1.80	0.140
Perceived social support (mean)^o^	9.66	9.68	9.65	0.592
Social activity (mean)^p^	2.82	2.71	2.92	0.047
**BMI (%)^q^**				
Under-weight	6	3	8	<0.001
Normal	27	27	28	
Over-weight	50	54	47	
Obese	17	17	17	
**SMOKING STATUS (%)**				
Ex-smoker	44	25	61	<0.001
Current smoker	46	67	29	
Never smoked	9	9	10	

*Values are percentages (numbers) or means*.

*^a^ Education score: composite score (0–3) using school leaving age, post school qualifications and highest qualifications; higher score denotes higher education level*.

*^b^ Income score: pre-tax weekly income (1–11 income categories ranging from 0 to $AUS926 or more)*.

*^c^ Self-rated health scores: excellent (0), very good (1), good (2), fair (3) poor (4)*.

*^d^ IADL scores: independent denotes able to shop, garden/do minor home maintenance, prepare meals and do housework on one’s own; dependent denotes needs help in at least one of shopping, gardening, preparing meals or housework*.

*^e^ Depression score: cut-off score for depression was a score of 5 or more on the PAS Depression scale (0–12), where 0 denotes no depressive symptoms and 12 denotes maximum number of depressive symptoms*.

*^f^ Falls requiring hospitalization*.

*^g^ Pain: daily pain (5), once or twice/week (4), once or twice/month (3), a few times a year (2), never (1)*.

*^h^ Urinary incontinence score: measures urge incontinence, where 0 is continent and 1 is incontinent*.

*^i^ Cognitive impairment score: derived from the Organic Brain Syndrome Scale (0–9), where 9 is more impaired*.

*^j^ Strain score: how often do you feel that you are under so much strain that your health is likely to suffer? Never (5), rarely (4), sometimes (3), frequently (2), very frequently (1). A high score indicates low strain*.

*^k^ Restful sleep score: how often do you feel really rested when you wake up in the morning? Most of the time (4), sometimes (3), rarely (2), never (1)*.

*^l^ Total physical activity score in last 2 weeks: type and frequency of activities scored 0–25, where 25 denotes more active*.

*^m^ Nutrition score: based in Australian Nutrition Screening Initiative, scores ranged from 0 to 19, where 19 denotes better nutrition*.

*^n^ Perceived social activity adequacy score: 1 is not enough or too much, 2 is about right*.

*^o^ Perceived social support score: 5 questions scored not true (0), partly true (1), certainly true (2), scores ranged from 0 to10*.

*^p^ Social activity score in last 2 weeks: nine activities scored as yes/no, scores ranged from 0 to 9*.

*^q^ BMI score: under-weight (<20), normal (20–25), over-weight (26–29), obese (30 and over)*.

### Sample

Table 1 also shows the baseline characteristics of the baseline participants. The mean participant age at baseline was 73.4 years (range 65–94 years) and 47% were male. A majority was married or widowed (90%) and around one-third lived alone. The mean number of medical conditions was 3.8 and most rated their health as good, very good, or excellent. The prevalence of depression was low (7%) and most experienced low strain and restful sleep. The table shows significant gender differences in these baseline characteristics: compared to men, women were on average slightly older and were much more likely to be widowed, to have lower education, and to report poorer health across the range of measures. Women had healthier lifestyles than men in terms of social activity, physical activity, and smoking status while men were healthier in terms of nutrition, fewer falls, and less urinary incontinence and they were less likely to be under-weight.

### Predictor variables

The predictor variables included in the study were organized into three blocks – socio-demographic, health, and lifestyle factors – in order to assess the potential for health promotion action in three main areas. The details of the specific measures are provided in the footnotes to Table 1.

*Socio-economic* factors were examined to identify target social groups for health promotion. This block included gender, age, marital status, education (a composite score based on highest education and age left school), income, country of birth, whether living alone or with others and whether participants had any living children.

*Health* factors focused on potential action areas in terms of specific disease prevention and treatment. This block included a tally of 33 self-reported medical conditions, self-rated health, depression [a score of 5 or more indicating significant depression on the psychiatric assessment scales (PAS) subscale of depressive symptoms; (41)], having had a fall requiring medical attention (42), pain, urinary incontinence (10), and degree of cognitive impairment (43).

*Lifestyle* factors focused on areas where enabling healthy ways of living could enhance health and well-being. This block included level of strain, restful sleep, level of physical activity, nutrition score, BMI, perceived adequacy of social activity, perceived social support, social activity level over the last 2 weeks (44), and smoking status.

All predictor variables were measured at baseline, irrespective of subsequent changes of health or other factors, thus providing an indication of the longer term consequences of the baseline health and social situations of older people rather than consequences of proximal changes through the course of later life.

### Outcome variable

*Aging well* was defined as continuing to live in the community (not in an institution) with at least good self-rated health (*good, very good*, or *excellent* as opposed to *poor* or *fair*), independence on all IADL components (shopping, gardening, and housework) adapted from Lawton et al. multilevel assessment instrument (44), *and* good psychological well-being (a positive affect score of 18 or better). The well-being measure corresponds to scoring 4 or 5 on most of the five items from the positive affect scale of Lawton et al. brief positive and negative affect measures (45). These measures are validated scales. In order to be classified as aging well, the participant had to continue to meet criterion on *all* of the indicators of aging well: good or better self-rated health *and* a positive affect score of 18 or better *and* independence in shopping, gardening and housework.

This composite aging well variable was measured at baseline and then at each of the subsequent survey rounds conducted every 2 years. While our interest is in positive outcomes in older age, the survival analysis technique requires us to conceptualize our primary outcome as “not aging well” in the tables. It is important to note that the biennial measurements do not identify various health and well-being changes that occur during the last interval period prior to leaving the state of aging well.

### Statistical analyses

Survival analysis was used to identify factors associated with the likelihoods of those who were aging well at baseline for continuing to age well throughout the 12 years of the study. For constructed scales, missing values on component items were pro-rated by the total on the rest of the items. Missing values were imputed on each construct (using mean-substitution). Multiple linear regression of the construct of interest on other relevant 1994 predictors within that construct’s block was carried out.

We conducted univariate analyses to identify correlates of not aging well. Variables found to be statistically significant (*p* < 0.10) in univariate analysis were included in multivariate models. Final models were determined via significance at *p* < 0.05 and deviance chi-square testing. Categorical variables were recoded into pair-wise comparison dummy variables: for example, country of birth with four categories (Australian, English, European, Other) was converted to three dummy variables with Australian acting as the reference group (English vs. Australian, European vs. Australian, Other vs. Australian). Marital status was converted to three dummy variables with married acting as the reference group (never married vs. married, divorced/separated vs. married, widowed vs. married). Effect Measure Modification was used to ascertain whether or not a predictor was specific to each gender. The final gender-based model therefore has predictors that are either specific for each gender or common to both genders.

## Results

### Baseline (1994) findings

At baseline (1994), all participants were living in the community. A total of *n* = 978 participants had data on all three aging well criteria (self-rated health, IADL, and positive affect scores) and 64.6% (*n* = 637) of these were classified as aging well. Of the total sample, *n* = 173 (17.7%) reported having fair or poor self-rated health, *n* = 169 (17.3%) were not fully independent IADL, and *n* = 149 (15.3%) had positive affect scores of 17 or less. Only *n* = 26 (2.7%) of the total sample had multiple disadvantages in terms of all three of these criteria, that is poor/fair self-rated health, dependent in IADL and low positive affect.

Table 2 shows the significant associations between demographic, health, and lifestyle variables and not aging well at baseline (1994). For these cross-sectional analyses, the criteria for aging well pertains only to the independence, self-rated health, and psychological well-being measures because all participants in the sample of course had already met the other criteria of being alive and living in the community. Being widowed and of European background were significant socio-economic threats to aging well while a high number of medical conditions and frequent pain were health threats to aging well. Whether participants were aging well or not was not associated with the socio-economic factors of gender, age, education, income, and living arrangements as well as the health factors of depression, falls, urinary incontinence, or cognitive impairment. The many lifestyle factors that were threats to aging well included low strain, restless sleep, low physical activity, poor nutrition, perceived inadequate social activity, inadequate social support, infrequent social activity, and being a current smoker (compared with being an ex-smoker). BMI did not impact on aging well at baseline.

**Table 2:** **Significant associations between demographic, health, and lifestyle variables and not aging well at baseline (1994) (*n* = 978)^a^**.

Variable	OR (95% CI)	*p*
**DEMOGRAPHIC**		
Widowed – married^b^	1.65 (1.19–2.28)	0.003
European – Australian^b^	1.70 (1.08–2.69)	0.023
**HEALTH**		
Tally of 33 medical conditions	1.22 (1.13–1.31)	<0.001
Pain	1.17 (1.06–1.29)	0.002
**LIFESTYLE**		
Low strain	0.75 (0.62–0.90)	0.002
Restful sleep	0.67 (0.54–0.84)	<0.001
Total physical activity	0.94 (0.91–0.98)	0.001
Nutrition score	0.89 (0.84–0.93)	<0.001
Perceived social activity adequacy	0.50 (0.34–0.74)	0.001
Perceived social support	0.81 (0.69–0.95)	0.008
Social activity	0.88 (0.80–0.96)	0.006
Smoking: ex-smoker-current smoker^b^	0.50 (0.30–0.83)	0.008

*^a^ Excludes the 22 cases for whom one or more aging well variables were missing*.

*^b^ Categories compared in the analysis*.

### Outcomes to 2006

By 2006, *n* = 410 had died, *n* = 50 were in residential aged care, *n* = 420 were still living in the community and of this latter group 398 had data on all three aging well criteria. Of these 42.5% (*n* = 169) were classified as aging well in that they were independent in activities of daily living and also had good self-rated health and psychological well-being.

### Survival analysis to 2006

The prospective analyses reported below were conducted only with the 637 respondents who were found to be aging well in the baseline survey. The multivariate survival analysis technique identified the most significant factors at baseline for people making a transition to a state of not aging well at some point over the 12-year study period.

Table 3 shows the final statistical models for the relative importance of independent baseline threats to aging well for women, men, and the total sample over the 12-year period after all factors had been taken into account. The table shows only predictors that reached significance in the final model. Variables that failed the bivariate criterion (*p* > 0.10) or lost significance in the multivariate model (*p* > 0.05) are not included in the table. The model for the total sample explained 22.2% of the variance in not aging well [χ^2^ (12) = 251.61, *p* = 0.000]. Older age was the only socio-demographic factor that remained significant in the final model while number of medical conditions was the only health variable remaining significant as a threat to aging well. Several of the lifestyle variables emerged as significant independent threats to aging well. These were poor nutrition, being under-weight (as compared to normal weight), and low levels of perceived social support. Sensitivity analyses were conducted by repeating the survival analyses on 10 randomly selected subsets consisting of 80% of the original data. In the combined gender models, all covariates except BMI remained significant in 10/10 of the analyses and BMI remained significant in 7/10, showing that this model was robust overall.

**Table 3:** **Final model for significant baseline threats to aging well over 12 years for men and women and the total sample^a^,^b^**.

Variable	Men not aging well		Women not aging well		Total not aging well	
	RR (95% CI)	*p*	RR (95% CI)	*p*	RR (95% CI)	*p*
**SOCIO-DEMOGRAPHIC**						
Age in years					1.08 (1.06–1.10)	<0.001
**HEALTH**						
Tally of 33 medical conditions					1.15 (1.09–1.21)	<0.001
Urinary incontinence			1.35 (1.06–1.71)	0.014		
**LIFESTYLE**						
Low strain	0.78 (0.68–0.89)	<0.001				
Perceived social activity adequacy	0.68 (0.51–0.91)	0.008				
Perceived social support					0.75 (0.66–0.86)	<0.001
Smoking: ex-smoker-current smoker^c^	0.61 (0.42–0.89)	0.010				
Nutrition score					0.95 (0.92–0.99)	0.017
BMI: normal-under-weight^c^			0.70 (0.49–1.00)	0.048^d^	0.48 (0.29–0.79)	0.004
Total physical activity			0.97 (0.95–0.99)	0.008		

*^a^*n* = 637 aging well at baseline*.

*^b^ Blank cells indicate that the baseline predictor failed the bivariate criterion (*p* > 0.10), lost significance in the multivariate model (*p* > 0.05) or was excluded on conceptual grounds*.

*^c^ Categories compared in the analysis*.

*^d^ Sensitivity analyses suggested that this borderline result for BMI was overall not a significant predictor*.

Several gender differences emerged as significant, independent influences on aging well outcomes. For women only, independent threats to aging well at baseline were urinary incontinence being under-weight (compared to normal weight) and low physical activity. For men only, independent threats to aging well were low strain, low perceived adequacy of social activity, and being a current smoker (as opposed to an ex-smoker) at baseline. In the male models, all covariates except being a smoker remained significant in 10/10 of the analyses and being a smoker remained significant in 6/10, showing that this model was reasonably robust. In the female models, urinary incontinence and total physical activity remained significant in 10/10 of the analyses, and BMI was significant in only 1/10, indicating that BMI was a marginal predictor in the final female model.

## Discussion

This study of community dwelling older Australians identified a number of threats to aging well that are potentially amenable for intervention. Our cross-sectional findings at baseline largely confirm that many socio-demographic, health, and lifestyle factors widely reported in previous studies have significant associations with different aspects of aging well. For this baseline sample of survivors in the community, nearly two-thirds were reported to be aging well in terms of self-rated health, independence in activities of daily living, and psychological well-being. Widows and European migrants were social groups found to be significantly at risk of not aging well. Other social determinants of health, notably income and education, were not found to be independently associated with aging well at baseline. A number of lifestyle factors were independently associated with aging well, including low strain, restful sleep, physical activity, nutrition, a range of social activity and social support factors and giving up smoking. Surprisingly, pain and the tally of medical conditions were the only significant health variables associated with not aging well. We suggest that the impacts of socio-economic resources and health for aging well mainly operate indirectly as predisposing influences on the lifestyle factors found to be directly associated with aging well in these cross-sectional associations.

The baseline cross-sectional associations suggest the importance of better understanding the potentially two-way relationships between lifestyle factors and aging well. However, as with comparable studies replete in the literature, cross-sectional data cannot disentangle the static and dynamic factors that are associated with aging well among those who were living in the community in 1994. Further, selection effects should be taken into account. While the overall burden of medical problems and level of pain were found to have understandable impacts, many acute health difficulties prevalent among older people surprisingly were not associated with aging well in our baseline analyses. Inevitably, the consequences of acute health problems cannot be identified very clearly in a cross-sectional community study when they are so severe as to result in death or institutionalization over a relatively short period of time. Alternatively, people may recover from less severe difficulties, manage them, or adapt in ways that enable them to stay independent, with good physical and mental health. Health conditions such as incontinence and cognitive impairment do remain important targets for intervention as they cause distress in older people and are associated with functional impairment.

By the end of the 12-year study period, those in the baseline sample with known outcomes were nearly equally divided between continuing survivors in the community and those who had died. Very few were living in residential aged care at any data collection and, among those who had died, only a third were known to have entered residential aged care beforehand. Among survivors continuing in the community throughout the study period, the majority still were aging well notwithstanding an average age of 73 years at baseline 12 years earlier. While experiences through later life of course are highly variable, a substantial number of people had continued for many years to experience good self-rated health, independence, and well-being.

The statistical procedure of survival analysis was applied to those who were aging well at baseline, in order to identify the most significant threats to aging well. Those classified as remaining as aging well consisted of those who in 2006 had remained living in the community with good self-rated health and positive affect and who were independent in activities of daily living (in comparison to the rest of the sample who over the course of the study had died, entered residential aged care, or were found to be aging unwell in 2006). This small group of what might be termed long term “successful agers” amounted to only 15% of those who were aging well 12 years earlier at baseline, although many more would have been aging well for some period prior to death or entry to residential care. While age and medical conditions remained as significant influences for aging well, the most important direct influences here (in contrast to the other outcomes) were a range of lifestyle factors including, nutrition, maintaining a healthy weight, and perceived social support.

Our findings regarding the role of behavioral and social factors for quality of life indicators are consistent with studies that have focused on specific aspects of aging well such as functional independence (17, 32). In a study of factors associated with the maintenance of exceptional good health in old age, Kaplan et al. (46) found that in a 10 year follow-up “thrivers” were more likely at baseline to have: higher income, lower psychological distress, never smoked, and moderate alcohol use. Our longitudinal findings may indicate causal factors underlying the earlier cross-sectional studies reporting associations between composite measures of successful aging and lifestyle factors, particularly not smoking, as influences in aging well (47, 48).

Our findings on gender underscore the importance of considering the different influences on aging well on older men and women. Important threats to aging well for men were lower strain, smoking, and lower perceived adequacy of social activity. Social connectedness appears to be important to men in determining their well-being in later life. However, the direct effect of low strain as a threat to aging well for men is somewhat surprising; other studies have found that allostatic load (physiological effects of chronic stress) is a predictor of mortality and low levels of stress can produce positive changes in the brains of older people (49). Perhaps low strain as measured in the current study is an indicator of low stimulation or even boredom, which has been associated with low cognitive function in older age (50). A core tenet of environmental psychology is that competency and well-being can be eroded by environments that are under-demanding as well as those that are over-demanding (51).

Threats to aging well important only for women need to be interpreted in the context that only 39% of them lived with a (potentially supportive) partner at baseline. For women only, these threats included incontinence, being under-weight, and lower physical activity – all of which are health and frailty influences on capacities for independent living, feeling well, and psychological well-being.

Comparing these threats to aging well with our previous findings on predictors of entry to residential aged care in the same sample we see that for men, the number of medical conditions and a healthy nutrition score (i.e., not psychosocial factors) were significant predictors of entry to residential aged care. For women, never having been married or widowed, IADL dependence, and under-weight BMI were significant, but urinary incontinence was not. These contrasting findings concerning predictors of two different outcomes (entry to residential aged care vs. not continuing to age well in the community in the same sample) highlight the importance of examining multiple outcomes for older people.

Our study design has several significant strengths. While much of the healthy and successful aging research reports cross-sectional findings, our prospective research design identifies the significance and relative importance of key baseline threats to aging well for older Australians. The use of baseline characteristics to predict outcomes is useful as the approach allows us to identify distal predisposing factors that may be amenable to change and therefore assist in the design of interventions for older people. For example, interventions that address urinary incontinence and low BMI are particularly salient for women while for men social interventions and addressing low stimulation are worthy of consideration.

The study also has limitations. As with all studies of older people, some important selection effects remain because we only examined at baseline those who had the good fortune of reaching later life in the community. This survival bias at the time would have been more pronounced among those who were at advanced ages when the study began in 1994. The study did not consider the influences of early life events and contexts on outcomes in old age; for example, a recent study by Westermeyer (22) found that psychological factors, such as good peer social adjustment in young age were predictive of successful aging. Similarly, Britton et al. (34) found that successful aging was influenced by early life health behaviors. In our view, the findings reported here are best understood as summary and directional findings that move beyond some of the problems of selection effects and other limitations of the existing research literature.

The current study did not examine the time varying influences of the baseline predictors and this creates the potential for information bias. However, our aim was to examine the distal predictors of aging well in order to identify early opportunities for health promotion. The next stage of our analyses will be to examine predictors and consequences of change between the 2-year intervals of our survey, thus more finely analyzing the time ordering of changes and identifying apparent causal pathways. Examining well-being transitions between the 2-year waves of the 12-year data collections should provide more precise indications of the proximal causes of changes in well-being in old age. We will identify “trajectories” and patterns of change, ways in which people enter and return from states of health and aging well; key factors in maintenance, improvement, or deterioration of healthy lifestyles; and ways in which individuals’ health and social changes influence the take-up of health and community services as well as entry to residential care. We are updating the data file to include further rounds of data collection through to 2010. These further analyses will aim to inform improvements in the efficacy and targeting of health promotion interventions including for those who are managing chronic illness and receiving community care.

Our long term MELSHA program has been contributing findings to two decades of developing health promotion initiatives in Australia (9). Baseline findings were disseminated and applied to initiatives led by the Victorian Health Promotion Foundation. Our earlier findings were considered by the Prime Minister’s Science Engineering and Innovation Council (PMSEIC, 5) which articulated an evidence-based vision for “an additional 10 years of healthy and productive life expectancy by 2050” (p. iv). Subsequent Australian initiatives in primary care, preventive health, self-care with chronic disease, and restorative approaches to aged care are focusing on the importance of lifestyle factors in multi-morbidity and aging, the potential for retaining and regaining independence in daily living, and the promotion of a broader behavioral and social health approaches to the needs of older people (52).

In conclusion, our study has shown that health actions and social resources in late life contribute to quality of life outcomes for older people. Such evidence reinforces the public case to invest in self-care and health promotion for older as well as younger people (9). It can drive attitudinal change amongst practitioners working with older people and society more generally, concerning the benefits of social and lifestyle interventions to improve health, independence, and well-being of the rapidly growing numbers of older people.

## Author Contributions

Hal Kendig and Colette J. Browning planned the study, supervised the data analysis, and wrote the paper. Shane A. Thomas and Yvonne Wells planned the study and contributed to revising the paper.

## Conflict of Interest Statement

The authors declare that the research was conducted in the absence of any commercial or financial relationships that could be construed as a potential conflict of interest.
